# Noonan syndrome with RAF1 gene mutations in a newborn with cerebral haemorrhage

**DOI:** 10.1186/s40001-022-00772-2

**Published:** 2022-08-11

**Authors:** Junwei Lan, Tianbao Zeng, Sheng Liu, Juhong Lan, Lijun Qian

**Affiliations:** Neonatology Department, Research Group in Lishui Municipal Central Hospital of Zhejiang Province, Lishui Hospital of Zhejiang University, Lishui, 323000 China

**Keywords:** Noonan syndrome, RAF1, cerebral haemorrhage, Newborn, 46, X, del (Y) (q12)

## Abstract

**Background:**

Noonan syndrome is an autosomal dominant genetic disorder that can occur in men and women and has a sporadic or family history. NS can lead to abnormal bleeding, but cerebral haemorrhage is rare. This is the first case of cerebral haemorrhage with a RAF1 gene mutation that originated in the neonatal period.

**Case presentation:**

This case presents a newborn with a RAF1 gene mutation resulting in NS complicated with an abnormality of chromosome 46, X, del (Y) (q12). In the course of treatment, the baby's breathing suddenly increased. After an MRI examination of the skull, haemorrhaging was found in multiple parts of the brain.

**Conclusions:**

After symptomatic treatment, the baby recovered well, but the main cause of cerebral haemorrhage was not found.

## Background

Noonan syndrome (NS) is a genetically heterogeneous disease that affects multiple systems. It is mostly an autosomal dominant disease, but there are a few cases of autosomal recessive inheritance [1]. The incidence of NS among live births is 1/2500 to 1/1000 [2]. The initial diagnosis of this disease can be made according to some special manifestations, including special facial features, heart malformation, bleeding and coagulation disorders, or skeletal deformities. Many genes play a role in NS, with approximately 19 genes currently involved [1]. Molecular tests for the four recognised genes in NS are now widely available. Mutations in PTPN11 account for 50%, KRAS accounts for 5%, SOS1 accounts for 15% and RAF1 accounts for 3–17% [3].

This is the first case of Noonan syndrome complicated with cerebral haemorrhage caused by RAF1 gene mutation in neonates.

## Case presentation

**Fig. 1:**
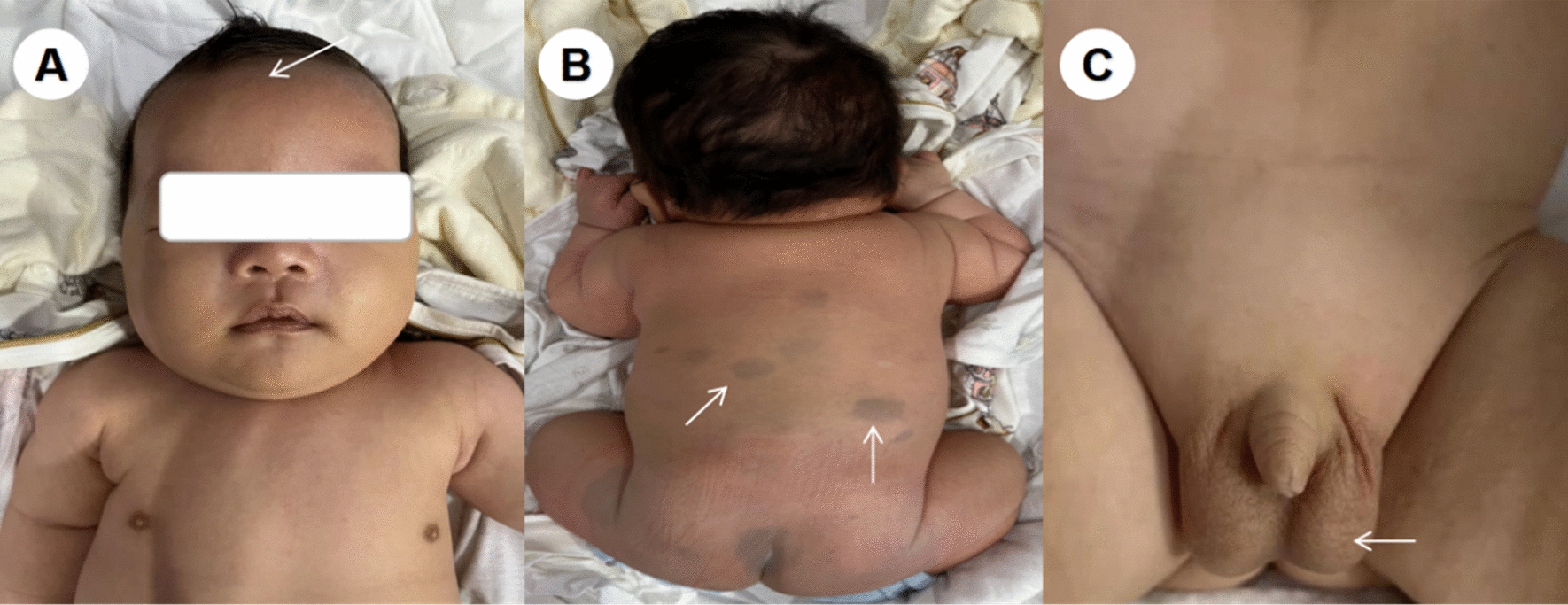
Appearance of the child. **A** Flat front fontanelle, high forehead, large head circumference, et al. **B** Blotchy moles on the back skin. **C** External genitalia are shown to be male, and the testicles are palpable on both sides

**Fig. 2:**
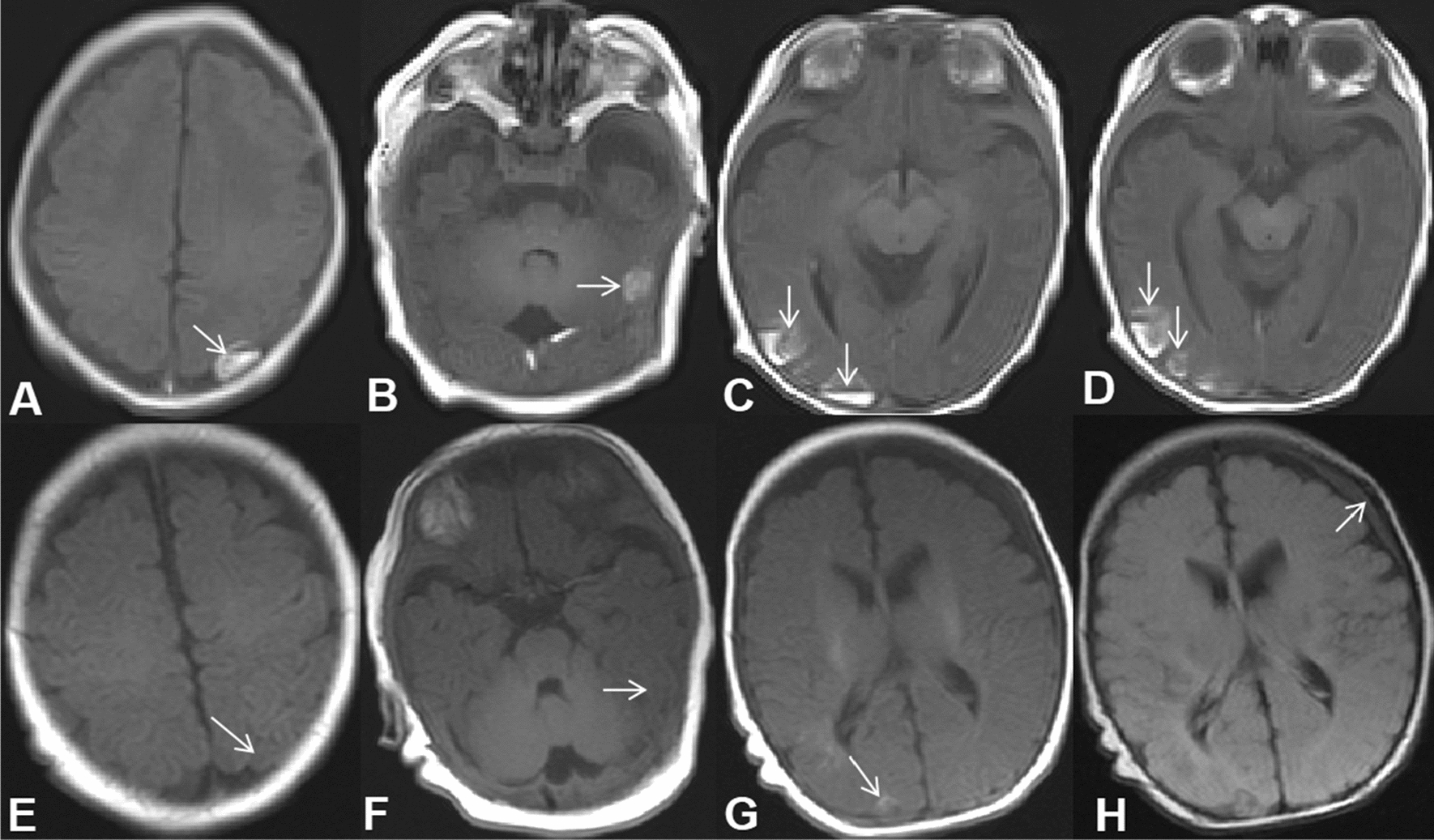
Magnetic resonance imaging (MRI) in newborn (**A**–**D** All images are T1-weighted) and follow-up (**E**, **F** T1-weighted images; **G**, **H** T2 flair images) 2 months later. **A** Left parietal haematoma. **B** Left temporal lobe haematoma. **C**, **D** Multiple haematoma in right occipital lobe. **E** The haematoma in the left parietal lobe has been absorbed. **F** The haematoma in the left temporal lobe has been absorbed. **G** The right occipital haematoma was absorbed more obviously than before. **H** Chronic subdural haematoma of left frontal region

**Fig. 3:**
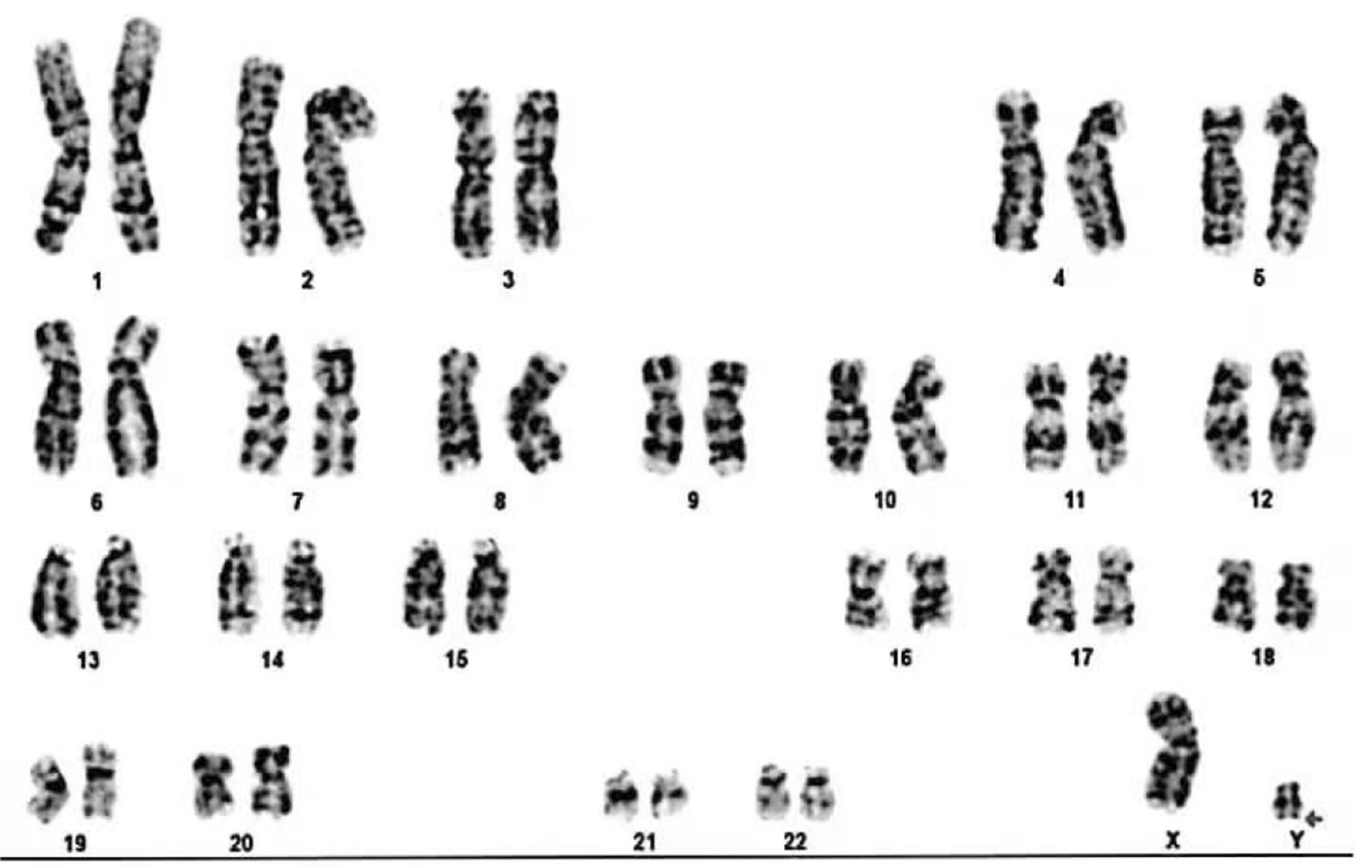
Karyotype analysis result was 46, X, del (Y) (q12)

**Fig. 4:**
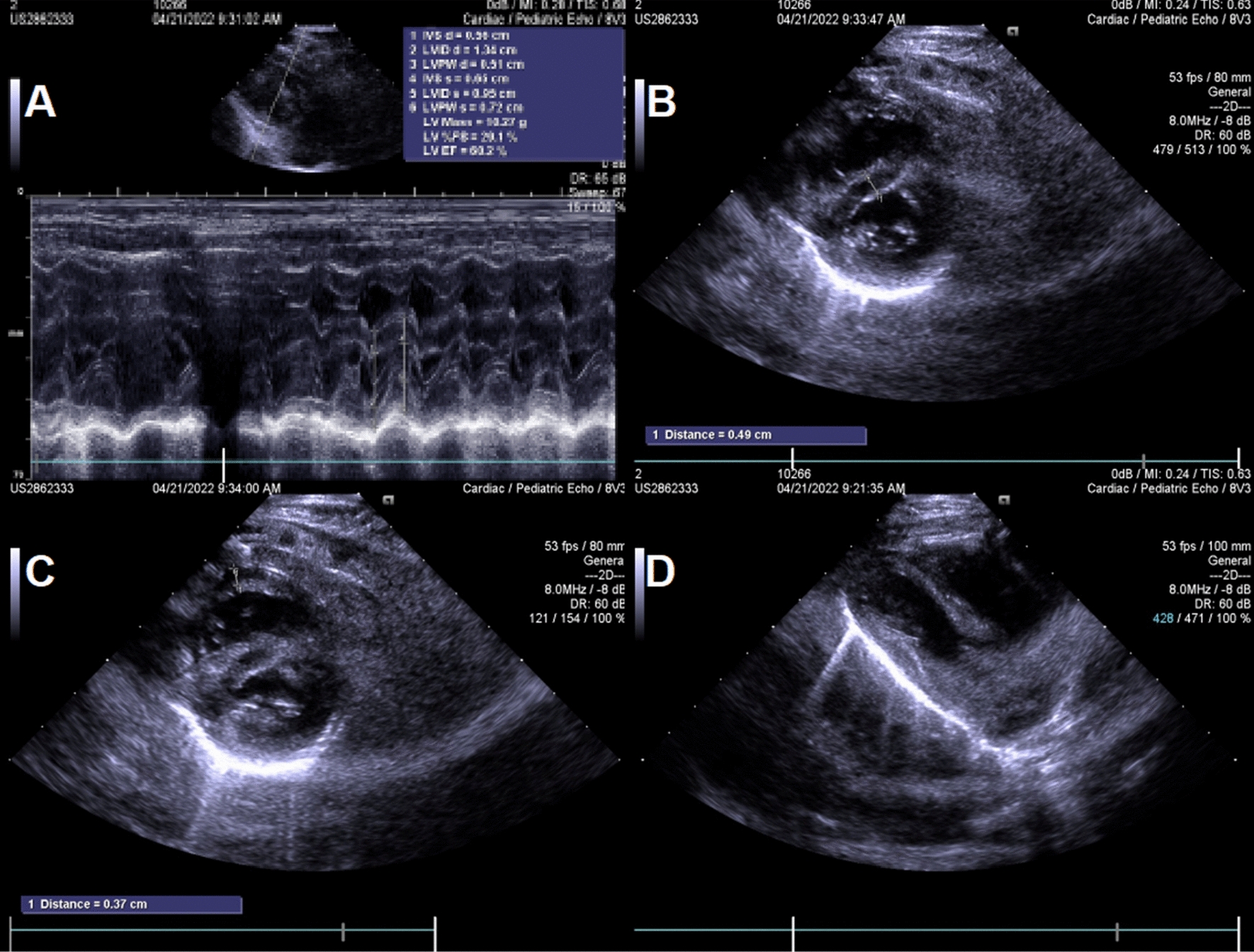
Two-dimensional echocardiogram in follow-up 2 months later. **A** Cardiac function EF: 60% and FS 29%. **B** Interventricular septum 5 mm. **C** Forearm of right ventricle 4 mm. **D** Stenosis of the left ventricle

The patient, a male newborn, was born for 40 min when he was admitted to our medical department because of “High-Risk Symptoms”. After 40 weeks of pregnancy, the mother gave birth naturally, and the whole process went smoothly. There was no complication of infection and abnormal blood glucose/blood pressure during pregnancy. The baby weighed 3900 g, and the length was 50 cm. The APGAR score was 9 points at 1 min and 10 points at 5 min. The physical examination report of the hospital is as follows: the vital signs of the baby were stable, and he has the appearance of a full-term infant; crying loudly, good response, flat front fontanelle, high forehead, large head circumference with a length of 38 cm; wide eye-distance, short nose, low-and-flat bridge of the nose, full nose-tip, low ear-position, short neck, blotchy moles on the back skin. Cardiopulmonary auscultation and limb muscle tone were normal. The baby had male external genitalia, the testes were descended, and there was no oedema on his dorsum of hands or feet (Fig. 1A–C). During the early foetal period, B-ultrasound examination showed polyhydramnios and bilateral pyelic separation of 6 mm. The results of amniocentesis showed that no obvious abnormality was found in the level of 320 bands of G-banded chromosomes in foetal amniotic fluid cells. B-ultrasound in late pregnancy suggested that amniotic fluid was still significantly elevated, head circumference was increased, and the femur length was shorter than expected. Prenatal whole-exome sequencing of the amniotic fluid showed that RAF1 mutation, p.Ser257Leu(c.770C > T)(PS2 + PS3 + PS4 + PM1 + PM2 + PP2),which was classified as a pathogenic mutation according to AMCG [4]. His parents and grandparents had no genetic metabolic disease, and his elder brother is currently 10 years old and developed normally. Laboratory examinations after admission were as follows: rapid hypersensitivity CRP 1.72 mg/L, white blood cell 11.9 × 10^9^/L, neutrophil granulocyte percentage 37.2%, lymphocyte percentage 44.5%, haemoglobin 154 g/L and platelet 245 × 10^9^/L. A plain film chest X-ray showed that two lung markings were slightly thickened, but the cardiac silhouette was not enlarged. Echocardiography suggested an atrial-septal defect (1.7 mm). Urinary system and cranial ultrasounds were unremarkable. On the third day after admission, the baby’s respiration rate increased significantly, reaching 80–90 breaths per minute. No abnormalities were found by lung auscultation, infection index analysis, pathogen culture or chest X-ray. Brain MRI showed areas of haemorrhage in temporal lobes, bilateral parieto-occipital lobes and bilateral paraventricular white matter (Fig. 2A–D). Coagulation/DD/AT-III reported APTT 41.9S, PT21.3S, D-D 0.53 mg/L, and AT-III was 59.0%. Coagulation factors II/V/VII/VIII/IX/X/XI were in the normal range, XII was 54.3%, and haemoglobin levels were lower than before. After oxygen, vitamin K1 and etamsylate supplementation, the head circumference did not increase, the anterior fontanelle remained flat and soft, and the breathing gradually stabilised. The patient was hospitalised for 10 days. His respiratory rate was approximately 50 times per minute, and his haemoglobin level did not decrease significantly when he was discharged. After discharge from the hospital, the karyotype analysis result was 46, X, del (Y) (q12) (Fig. 3). The phenotype of the neonatal whole blood exon gene showed that the phenotype of the RAF1 mutation, p.Ser257Leu(c.770C > T) (Table 1), which was a pathogenic variation. These findings reaffirmed the previous diagnosis of NS in this patient. Two months after discharge, the baby came to the hospital again for follow-up and showed good growth and development, and his weight had reached 6000 g. Head MRI reexamination reported that the cerebral haemorrhage had improved significantly (Fig. 2E–H). Echocardiography showed ventricular hypertrophy (interventricular septum 5 mm, posterior wall of the left ventricle 5 mm, the forearm of right ventricle 4 mm) (Fig. 4). The patient is being followed up at present.

**Table 1:** RAF1 mutation, p.Ser257Leu(c.770C > T) [4]

PTPN11	SOS1	SOS2	RAF1	RIT1
KRAS	NRAS	RRAS	HRAS	SHOC2
LZTR1	A2ML1	BRAF	MAP2K1	MAP2K2
NF1	SPRED1	RASA1	PPP1CB

## Discussion

NS was first discovered and named by Jacqueline Anne Noonan in 1962. The disease is associated with multiple gene mutations in the RAS-MAPK signalling pathway. These genes play a role in the regulation of tissue growth, cell division, metastasis and differentiation [5]. The RAF1 gene is located on chromosome 3, and most of the RAF1 mutations associated with NS are clustered in two regions, namely, conserved regions 2 and 3. Mutation in the RAF1 gene has been connected with a variety of maladies covering hypertrophic cardiomyopathy, mental retardation, short stature, and Noonan-like skin features associated with freckle syndrome (multiple freckles, nevi cafe-au-lait, multiple nevi). More than 90% of NS patients with mutations in conserved region 2 of RAF1 suffer from severe hypertrophic cardiomyopathy, resulting from abnormal ERK5 and MEK1/2 signalling [6].

In this case, the newborn in the foetal period repeatedly found that the amniotic fluid increased significantly, and the biparietal diameter widened significantly. We performed amniocentesis and found a mutation in the RAF1 gene C. After birth, a physical examination showed a large head circumference (38 cm), and the following abnormal signs appeared at the same time: wide eye-distance, short nose, low bridge of the nose, full nose-tip, low ear-position, short neck, and spot moles on the back (Fig. 1A, B). The above clinical manifestations are consistent with the clinical characteristics of NS in the neonatal period [7]. Two months after the patient’s birth, echocardiography showed myocardial hypertrophy (Fig. 4), and the full exogenous gene report of blood samples was still RAF1 gene mutation C. 770C > T (p. S257Lp. Ser257Leu) (Table 1). Again, the diagnosis of NS was confirmed. Because of coagulation dysfunction, patients with NS are prone to ecchymosis or bleeding, and the bleeding rate often reaches 30–65% [5]. The most common cause is a factor-XI deficiency; other causes include the decreased activity of factors VIII and XII, vWF deficiency, thrombocytopenia and platelet functional defects. There are also rare causes, such as factor XIII deficiency, but the most common causes of bleeding are not clear [8, 9]. Although NS causes a high rate of bleeding, reports of intracerebral haemorrhage are rare. Tang found that NS patients were complicated with moyamoya disease and cerebral haemorrhage [10]. Dineen reported two cases of NS complicated by subarachnoid haemorrhage, the cause of which was a cerebral aneurysm [11]. Orrego-González reported a case of a 12-year-old girl who was an NS patient with PTPN11 mutation complicated by intracerebral haemorrhage [12], but no specific cause was found. Mussa recently found a newborn with NS complicated by cerebral haemorrhage due to PTPN11 mutation. In addition, the causes included that the baby suffered from neonatal alloimmune thrombocytopenia at the same time [13]. On the other hand, our patient had a sudden increase in respiration on the third day after admission, and there were no symptoms, such as fever, vomiting, apnoea, convulsions, neurasthenia, changes in muscle strength or limb tension. No abnormalities were found by chest X-ray, related infection indicators, or pathogen culture. Finally, cranial MRI revealed multiple haemorrhages in the brain. A brain ultrasound examination was performed on the second day after admission. However, brain ultrasound was relatively sensitive to ventricular haemorrhage and less sensitive to temporal–parietal haemorrhage. After the discovery of cerebral haemorrhage, we carried out the following coagulation function tests: PT, APTT and DD were normal, and coagulation factors II/V/VII/VIII/IX/X/XI were in the normal range. XII 54.3% was slightly lower than normal. Although XII can cause prolonged APTT, XII has no risk of bleeding, which instead causes blood clots [14, 15]. We also performed a routine blood test on the baby and found that the platelet count was normal and that haemoglobin did not decrease significantly. After taking vitamin K1 and haemostatic supplements in addition to oxygen inhalation, the children's breathing gradually stabilised. When discharged from the hospital, routine blood examination showed that haemoglobin decreased slightly compared with before.

Generally, blood vessels, platelets and coagulation factors work together to maintain the balance of bleeding and coagulation in the human body. The labour process is smooth, and the possibility of cerebral haemorrhage caused by labour injury was small. Two cases of NS due to FAF1 gene mutation can lead to intracranial vascular malformation [16, 17] (Table 2); Because RAF1 plays an important role in vascular matrix production [18] and vascular immune protection [19], mutations in RAF1 gene can lead to cerebral vascular malformations, resulting in cerebral haemorrhage. Since our case had multiple cerebral haemorrhage, the possibility of cerebrovascular malformation was small. Meanwhile, the child's parents refused CTA examination due to radiation. The platelet count of the patient was normal on multiple evaluations, and since the patient did not have petechiae and ecchymoses, so bleeding caused by platelet self-count or dysfunction was not considered. The patient was routinely supplemented with vitamin K1 after birth. Coagulation function was normal, and the function of most coagulation factors was in the normal range. However, factor XIII was not monitored in the patient. Factor XIII deficiency can cause massive uncontrolled bleeding. For this reason, infusion of cryoprecipitate or fresh frozen plasma containing cryoprecipitate is required for effective haemostasis [6]. Therefore, it is not convincing to think that the lack of coagulation factors leads to bleeding in the patient. We cannot ignore the bleeding caused by a lack of vWF because abnormal vWF can cause platelet GPIIb/IIIa receptor-mediated platelet dysfunction, resulting in bleeding [20]. Limited to hospital conditions, we did not measure the vWF factor of the patient. In summary, the cause of the haemorrhaging in this baby is still unclear.

**Table 2:** Previously reported cerebrovascular abnormalities in Noonan syndrome with RAF1 mutation

Abnormality	Age	Gender	Cardiac	Noonan findings	Other	Reference
Chiari malformation type I	6y	Male	—	Special face, short stature, cryptorchidism RAF1 mutation	—	Zarate et al. [16]
Subdural haematoma and associated with cavernous haemangiomata	4m	Male	—	Special face, short stature RAF1 mutation	—	Hartill et al. [17]

Chromosomal examination of this newborn with NS revealed 46, X, del (Y) (q12) (Fig. 3). Chromosomal abnormalities can cause gonadal dysgenesis, azoospermia, etc. [21]. As shown in Fig. 4, the child's external genitalia are shown to be male, and the testicles are palpable on both sides (Fig. 1C). The baby needs further follow-up, in which genital development should be considered. If necessary, testicular B-ultrasound, sex hormones and other examinations need to be performed.

## Follow-up

Two months after discharge, the follow-up showed that the growth and development of the child were good. Head MRI examination suggested that the cerebral haemorrhage had recovered. However, the child developed hypertrophic cardiomyopathy, which was consistent with Mussa A, who discovered the RAF1:c.770C > T (Ser257Leu) mutation [6], but the patient [6] developed myocardial hypertrophy and pulmonary hypertension during the neonatal period, resulting in death during the neonatal period. However, no effective treatment has been found for myocardial hypertrophy caused by RAF1, and its clinical study is currently underway [22]. Therefore, the child needs close follow-up in the outpatient clinic, and drug therapy or even cardiac replacement can be tried if necessary.

## Conclusion

With the development of molecular biology diagnostic techniques, the diagnosis rate of NS in the foetal and neonatal periods will be significantly increased. Because of the high rate of abnormal haemorrhaging in NS, intracranial haemorrhage should be considered when children with NS have abnormal symptoms, especially those of the nervous system. In the initial stage of treatment, the children should be examined by B-ultrasound and MRI. Because of the high probability of myocardial hypertrophy caused by RAF1 mutation, B-mode ultrasonography should be performed as soon as possible after discharge to prevent misdiagnosis and missed diagnosis of hypertrophic cardiomyopathy. Neonatal cerebral haemorrhage can lead to a high mortality rate, especially multiple cerebral haemorrhages. The development of the baby's parents and his elder brother was normal, and no abnormal cases of NS were found in his family. Therefore, the parents of the baby suffer from tremendous mental pressure. With this in mind, in addition to timely clinical diagnosis and effective treatment, patients need compassionate care and close communication from our health care workers. The child's cerebral haemorrhage is in good condition, but cardiac hypertrophy is still a considerable challenge for the family. However, we hope that for such a rare, difficult disease, through the joint efforts of medical staff and family members, as much as possible to make the child healthy and enjoy happy growth.

## Data Availability

All data generated or analysed during this study are included in this published article.
